# Are clinical trajectories in obstructive hypertrophic cardiomyopathy mutation-specific? A systematic review and meta-analysis of *MYH7* vs. *MYBPC3*

**DOI:** 10.3389/fcvm.2026.1752303

**Published:** 2026-04-29

**Authors:** Yunqing Li, Jin Yu

**Affiliations:** Department of Cardiology, Xijing Hospital, The Fourth Military Medical University, Xi'an, China

**Keywords:** genetic mutation, genotype–phenotype, hypertrophic cardiomyopathy, meta-analysis, obstructive HCM

## Abstract

**Background:**

The clinical course of obstructive hypertrophic cardiomyopathy (HCM) is highly variable. Whether this heterogeneity reflects mutation-specific clinical trajectories remains a pivotal question for prognostication and personalized management.

**Objectives:**

This study aimed to determine if pathogenic sarcomeric variants in *MYH7* and *MYBPC3* are associated with distinct clinical pathways in obstructive HCM, by comparing their associations with age at presentation, severity of hypertrophy, left ventricular outflow tract (LVOT) obstruction, and arrhythmic outcomes.

**Methods:**

We conducted a systematic review and meta-analysis following PRISMA 2020 guidelines. Four databases were searched up to October 2025 for cohort or registry studies reporting on sarcomeric variants and quantitative phenotypes in obstructive HCM. Data were extracted independently in duplicate, and effect estimates were pooled as odds ratios (OR), mean differences (MD), or hazard ratios (HR) with 95% confidence intervals (CI).

**Results:**

Ten studies comprising 192,361 participants were included, encompassing a large population sequencing cohort (UK Biobank/Mass General Brigham) of 184,511 and nine clinical/registry cohorts totaling 7,850. Carriers of *MYH7* variants presented with a more obstructive and arrhythmogenic phenotype, demonstrating higher odds of LVOT obstruction (pooled OR range: 1.95–4.30) and a 70% increased risk of incident atrial fibrillation (HR: 1.7, 95% CI: 1.1–2.6). In contrast, carriers of *MYBPC3* variants, while often presenting later, faced a significantly elevated risk of progressive systolic dysfunction (adjusted HR: 2.53, 95% CI: 1.09–5.82). *MYH7* was also associated with greater interventricular septal thickness (MD: 1.30 mm, 95% CI: 0.06–2.54). Substantial heterogeneity (*I*^2^ = 80.7%) was observed, reflecting real-world differences in phenotype ascertainment.

**Conclusions:**

This analysis confirms that obstructive HCM follows mutation-specific clinical trajectories. Genotype provides critical prognostic insights, delineating an *MYH7* driven path of early obstruction and arrhythmia from an *MYBPC3* associated trajectory of long term systolic decline. These findings support the integration of genetic data into personalized surveillance strategies.

## 1 Introduction

Hypertrophic cardiomyopathy (HCM) is a common inherited myocardial disorder with population prevalence near 1:500 and a spectrum that ranges from silent left-ventricular hypertrophy to heart failure and sudden cardiac death (SCD) (1, 2). Structural abnormalities appear in 25% of first-degree relatives, and roughly 60% of patients carry mutations in sarcomeric or sarcomere-related genes. Among mutation-positive cases, *MYH7* and *MYBPC3* account for nearly 75%, while variants in *TNNT2*, *TNNI3*, *TPM1*, *MYL2*, *MYL3*, and *ACTC1* contribute 1%–5%. Despite the autosomal dominant pattern, up to 40% of patients remain genotype-negative, reflecting additional molecular mechanisms underlying HCM’s variable expression and penetrance (3). The obstructive phenotype, defined by dynamic left-ventricular outflow tract (LVOT) gradients, drives symptoms, exercise limitation, atrial arrhythmia, and higher procedural intervention rates, making obstruction a central clinical problem in HCM care (4, 5). Genetic studies over three decades have established that the majority of pathogenic variants reside in sarcomeric genes, predominantly *MYBPC3* and *MYH7*; however, their penetrance and expressivity exhibit considerable heterogeneity across families and populations (1, 6). This variability arises from single-gene effects, compound variants, and background polygenic risk, together with non-genetic modifiers, which produce marked heterogeneity in age at onset, maximal wall thickness, LVOT obstruction, and arrhythmic risk (7, 8). Published cohorts and registries yield inconsistent gene-specific signals: some series report more severe, earlier phenotypes with *MYH7* variants, while others find overlapping morphology and outcomes between *MYH7* and *MYBPC3* carriers (2, 7, 9). This inconsistency limits the clinical utility of genotype for individual risk stratification but highlights the need for meta-analytic approaches to clarify genotype-phenotype patterns and effect sizes across studies. Pooling genotype-phenotype data can help resolve whether genotype predicts age at diagnosis, likelihood or severity of LVOT obstruction, septal thickness, and SCD risk outcomes that influence surveillance, lifestyle advice, and therapeutic choice, including targeted myosin inhibitors and timing of invasive septal reduction (1, 4, 10). The primary objective was to quantify associations between sarcomeric genotypes and key phenotypes (age at onset, LVOT obstruction, septal thickness, SCD). Secondary objectives were to evaluate gene-specific differences (*MYH7* vs. *MYBPC3*), assess heterogeneity sources, and appraise implications for clinical management.

## 2 Primary objective

Quantify association between pathogenic/likely pathogenic sarcomeric gene variants and presence of LV outflow tract obstruction.

Secondary objectives

Association with interventricular septal thickness (IVS), age at diagnosis, family history, arrhythmia events.

Subgroup associations by gene (*MYH7*, *MYBPC3*, *TNNT2*, *TNNI3*, others) and variant type (truncating vs. missense).

## 3 Methodology

This systematic review and quantitative synthesis followed PRISMA 2020 guidelines and focused on evaluating genotype–phenotype associations in obstructive hypertrophic cardiomyopathy (HCM). The review was designed to integrate clinical, imaging, and genetic data across cohort and registry-based studies that investigated pathogenic or likely pathogenic (PLP) sarcomeric variants, including *MYH7*, *MYBPC3*, *TNNT2*, and *TNNI3*, in relation to left ventricular outflow tract obstruction (LVOTO), septal morphology, and arrhythmia outcomes.

## 4 Search strategy and study selection

A comprehensive literature search was conducted in PubMed, Scopus, Embase, and Web of Science from inception to October 2025. Search terms combined MeSH and free-text keywords related to “hypertrophic cardiomyopathy,” “obstructive,” “genotype–phenotype,” “*MYH7*,” “*MYBPC3*,” and “sarcomeric mutation.” Only peer-reviewed cohort, registry, or population-based genetic association studies with defined genotypic subgroups were included. Reference lists of key articles were manually screened to ensure completeness.

## 5 Inclusion and exclusion criteria

Eligible studies included adult or adolescent patients with genetically confirmed HCM in whom genotype-positive (G+) and genotype-negative (G−) or gene-specific subgroups were directly compared. Quantitative reporting of structural phenotypes (e.g., interventricular septal thickness, LVOT gradient) or clinical outcomes (arrhythmia, systolic dysfunction, or heart failure events) was required. Only studies using validated variant interpretation systems (ACMG/ClinVar or institutional pathogenicity frameworks) were accepted. Exclusion criteria comprised studies without genotyping, case reports, family-only descriptive pedigrees, single-variant mechanistic studies, or purely experimental work.

Obstructive HCM was defined as the presence of a LVOT gradient ≥30 mmHg at rest or with provocation, according to current guideline-consistent haemodynamic criteria in the majority of clinical and registry cohorts. Eligible studies used existing contemporary guideline definitions which defined thresholds as ≥30 mmHg and ≥50 mmHg to measure resting and provoked gradients. The SHaRe registry analysis (11) and the UK Biobank study (12) relied on ICD/HER code-based HCM ascertainment and did not uniformly apply a documented gradient threshold; these eligible studies quantified LVOT-related phenotypes as secondary outcomes rather than as primary inclusion criteria.

During study selection, individual study was assessed whether it (a) explicitly enrolled patient meeting a haemodynamic definition of obstructive HCM (gradient ≥30 mmHg), or (b) reported obstruction-related phenotypes as primary outcome in a well-characterized HCM cohort with documented characterization of obstructive subsets. Studies that enrolled mixed obstructive and non-obstructive HCM populations without providing obstruction-stratified subgroup were excluded. This heterogeneity in operational definitions was pre-specified as a potential source of between-study variance, and LVOT obstructive definition was incorporated as a stratification variable to explore heterogeneity, using *I*^2^ statistics.

## 6 Data extraction and synthesis

**Figure 1:**
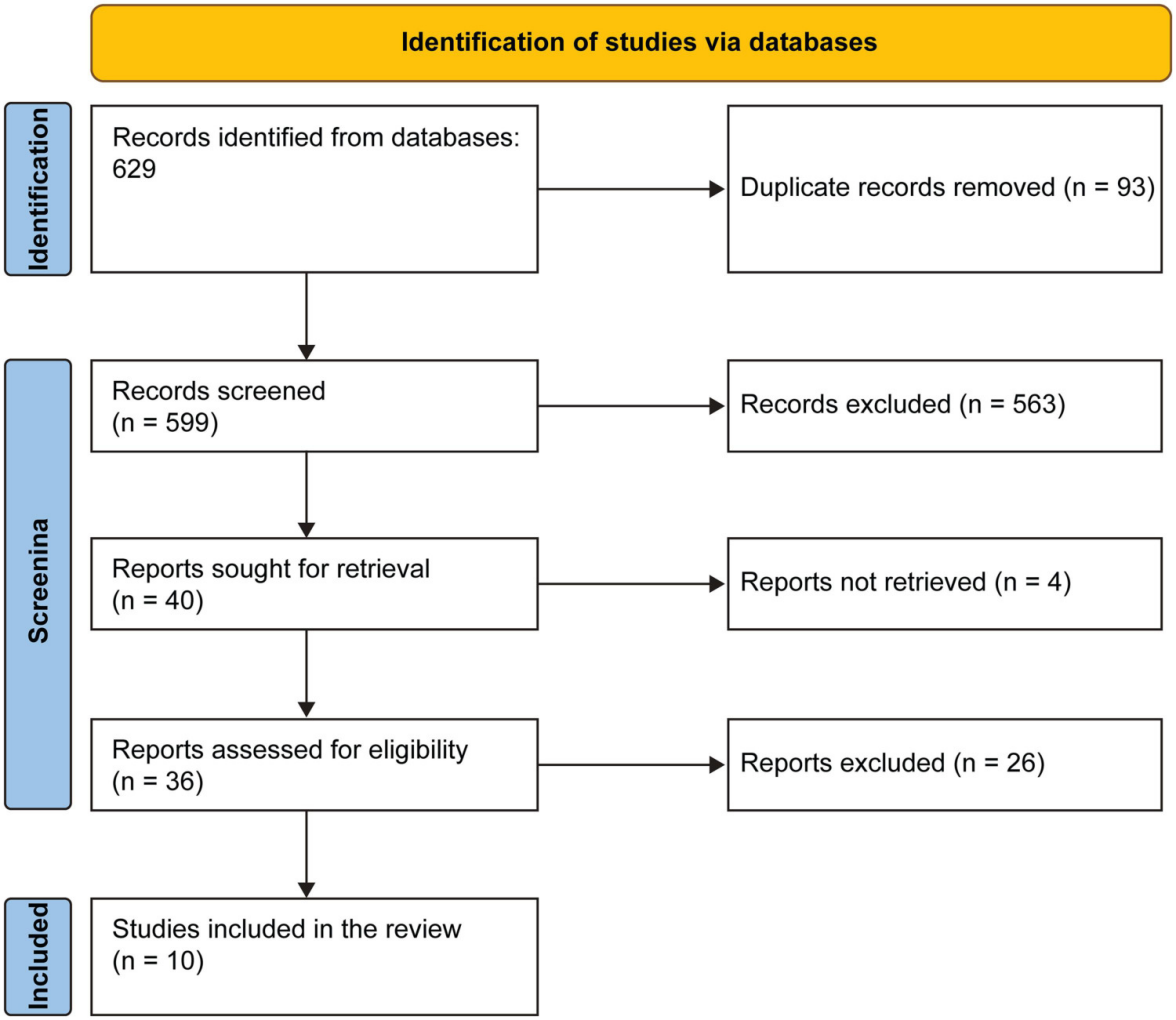
PRISMA flow diagram of the study selection process.

Two reviewers independently extracted data on design, cohort size, genotype distribution, variant type (truncating vs. missense), and quantitative outcomes. Results were harmonized for meta-analytic comparison using odds ratios (OR), mean differences (MD), or hazard ratios (HR) with corresponding 95% confidence intervals (CI). Heterogeneity was evaluated using *I*^2^ statistics and fixed-effect pooling was used when between-study variance was low (*I*^2^ < 50%). Funnel plots were visually inspected for asymmetry. The search strategy and screening process are detailed in the PRISMA flow diagram (Figure 1). The baseline characteristics of the included studies and a summary of genotype-phenotype associations are presented in Tables 1 and 2, respectively. Detailed study-level data for the primary outcome (LVOT obstruction), continuous outcome (IVS thickness), and heterogeneity exploration are provided in Appendix Tables 5, 6 and 7, respectively.

**Table 1:** Baseline characteristics of included studies.

Study (ref)	Country/setting	Design	Total N
Velicki et al. (2)	Multicentre UK/Europe	Cohort, genotype-positive comparison (single-gene groups)	63 (final analysis)
Beltrami et al. (9)	European centres (likely Italy)	Cohort, long-term follow-up, gene comparison	402 reported
Li et al. (10)	Multicentre (Canada + collaborators)	Cohort with follow-up; genotyped probands	558
Helms et al. (11)	Multi-national registry (SHaRe)	Registry cohort analysis	∼4,756 genotyped patients (SHaRe)
Biddinger et al. (12)	UK Biobank and Mass General Brigham	Population/biobank genetic association	184,511 sequenced/genotyped (subset analysed); HCM cases *n* = 204
Höller et al. (14)	Austria, Medical University of Graz	Prospective registry	Noted per-analysis: *MYBPC3 n* = 39, *MYH7 n* = 18
Schoonvelde et al. (16)	Multicentre	Cohort comparing genotype-positive and genotype-negative HCM	422
Guo et al. (15)	Noted in paper	Cohort of obstructive HCM patients	402 obstructive HCM patients
Lee et al. (13)	SHaRe registry, international	Registry cohort; incident AF analysis	1,040 adult HCM patients without baseline AF
Viswanathan et al. (7)	USA (single centre with collaborators)	Cohort/family series with symptomatic, asymptomatic carriers and noncarriers	150 (80 symptomatic + 35 asymptomatic carriers + 35 non-carriers)
Study (ref)	N mutation positive	N mutation negative	Genes/groups analysed
Velicki et al. (2)	63 (*MYBPC3 n* = 48; *MYH7 n* = 15)	0	*MYBPC3* vs. *MYH7*
Beltrami et al. (9)	Available by gene in full text	Noted comparisons to *MYH7*	*MYBPC3* vs. *MYH7* comparisons
Li et al. (10)	198 (35.4%)	∼360	Sarcomeric pathogenic vs. non-pathogenic; subgroup *MYH7*/*MYBPC3* reported
Helms et al. (11)	*MYBPC3* carriers *n* = 1,316 (truncating 1,047; nontruncating 191)	Many other genotypes and G− patients in registry (not primary here)	*MYBPC3* detailed (truncating v nontruncating)
Biddinger et al. (12)	ClinVar/PLP rare variants in core genes (gene-level carriers small)	Majority non-carriers in biobank	Core sarcomeric genes including *MYBPC3*, *MYH7*
Höller et al. (14)	*MYBPC3 n* = 39; *MYH7 n* = 18	Not primary comparator	*MYBPC3* vs. *MYH7*
Schoonvelde et al. (16)	G+ proportion ≈54% (so ∼227)	G− proportion ≈46% (so ∼195)	Genotype-positive vs. genotype-negative comparisons
Guo et al. (15)	*MYH7 n* = 94; *MYBPC3 n* = 76; mutation-negative *n* = 212	212 mutation-negative	*MYH7* vs. *MYBPC3* vs. mutation-negative
Lee et al. (13)	*MYH7 n* = 296; *MYBPC3 n* = 659; thin filament *n* = 85	Some genotype-negative or other genes not listed here	Sarcomeric gene groups (*MYH7*, *MYBPC3*, thin filament)
Viswanathan et al. (7)	Symptomatic carriers: *MYBPC3* = 59; *MYH7* = 31 (reported)	35 non-carriers	18-gene panel; per-gene counts reported

Study (ref)	Variant type (as reported)	Mean follow up (years)	Key phenotypes/outcomes reported
Velicki et al. (2)	Pathogenic variants (authors state “confirmed single pathogenic mutations”); variant-type detail likely in supplement	Cross-sectional (no longitudinal follow-up reported)	Symptoms, family history, AF prevalence, systolic anterior motion, mitral leaflet abnormalities, *E*/*e*′
Beltrami et al. (9)	Pathogenic variants per methods	Long-term follow-up (reported in text)	Obstructive phenotype at presentation, LVEF, new severe LV systolic dysfunction, Cox model for systolic dysfunction
Li et al. (10)	Pathogenic/likely pathogenic per lab methods	6.3 years (mean)	Septal reduction procedures, AF, combined heart-failure end point (time-to-event)
Helms et al. (11)	Adjudicated pathogenic calls by SHaRe	Variable by registry entry (time-to-event analyses used)	Composite outcomes (SCD, HF events, LVAD/transplant, AF, stroke, death), wall thickness distributions
Biddinger et al. (12)	ClinVar/curated LOF; ACMG-informed curation	N/A for classic follow-up; cross-sectional case ascertainment	Gene-level ORs for HCM and phenotype by ICD/EHR codes (not echocardiographic gradients)
Höller et al. (14)	Variant calls per registry	Follow-up variable; imaging metrics emphasized	IVS thickness and myocardial deformation (strain) by gene
Schoonvelde et al. (16)	Variant classification per methods	Reported follow-up in full text	Baseline phenotype and CV risk factors by genotype status
Guo et al. (15)	Pathogenic calls per methods; regression analyses adjust covariates	Cross-sectional with multivariable regressions	Mitral leaflet length, IVS thickness, late gadolinium enhancement; *MYH7* linked to more severe disease
Lee et al. (13)	Pathogenic/likely pathogenic per SHaRe	Mean follow-up 7.2 years	Incident AF events (198 incident AF events). Adjusted HRs for incident AF by gene. numbers will be guessed according to this reference lit: 1.
Viswanathan et al. (7)	Mutation calls reported; details in paper	Cross-sectional/follow-up elements	Echocardiographic phenotype comparisons; open data in tables

**Table 2:** Summary of Key genotype-phenotype associations and effect estimates.

First Author	Key genotype groups (*N*)	Main numeric phenotype findings (reported)	Reconstructed counts or effect estimates (as reported)
Velicki et al. (2)	*MYBPC3 n* = 48; *MYH7 n* = 15	AF prevalence: *MYBPC3* 35% vs. *MYH7* 60% (*p* = 0.085). Systolic anterior motion (SAM): *MYBPC3* 10% vs. *MYH7* 33% (*p* = 0.025). Mitral leaflet abnormalities: *MYBPC3* 19% vs. *MYH7* 40% (*p* = 0.039). Mitral annulus calcification: 0% vs. 20% (*p* = 0.001). *E*/*e*′ ratio (mean ± SD): *MYBPC3* 8.8 ± 3.3 vs. MYH7 13.9 ± 6.9.	Reconstructed counts using group Ns: AF *MYBPC3* 17/48, AF *MYH7* 9/15. SAM *MYBPC3* 5/48, SAM *MYH7* 5/15. MLA *MYBPC3* 9/48, MLA *MYH7* 6/15. MAC *MYBPC3* 0/48, MAC *MYH7* 3/15. *E*/*e*′ values given directly.
Li et al. (10)	Genotyped probands total 558. Genotype positive 198; genotype negative ∼360	Genotype-positive status associated with earlier age at diagnosis (mean/median 39 vs. 48 years) and greater family history (53% vs. 20%). In multivariable survival models, genotype-positive associated with combined heart-failure end point.	Adjusted hazard ratio for combined heart-failure end point: HR: 4.51 (95% CI: 2.09–9.31), *P* < 0.001. Mean follow-up 6.3 years.
Helms et al. (11)	SHaRe *MYBPC3* carriers *n* = 1,316 (truncating 1,047; nontruncating 191)	Composite outcomes (SCD, severe HF, LVAD/transplant, LVEF <35%, AF, stroke, death) reported by variant type. No significant difference between truncating and nontruncating *MYBPC3* carriers for composite outcome.	Time-to-event analyses shown; no pooled HR reported comparing truncating vs. nontruncating. Paper reports Kaplan–Meier curves and statistical tests without a single pooled effect to extract here.
Beltrami et al. (9)	Total reported cohort *n* = 402. Gene-specific Ns in full text	At presentation, obstructive phenotype: *MYBPC3* 15% vs. *MYH7* 26% (*p* = 0.005). LVEF: *MYBPC3* 66 ± 8% vs. *MYH7* 68 ± 8% (*p* = 0.03). New severe LV systolic dysfunction (LVEF <50%): *MYBPC3* 15% vs. *MYH7* 5% (*p* = 0.013).	Cox multivariable for new severe systolic dysfunction: *MYBPC3*-positive HR: 2.53 (95% CI: 1.09–5.82), *P* = 0.029.
Viswanathan et al. (7)	Symptomatic carriers: *MYBPC3* 59; *MYH7* 31. Asymptomatic carriers and non-carriers also included	Many measured echocardiographic parameters showed no statistically significant differences across gene groups in that cohort.	Counts and tables available in full text. Summary conclusion: no large genefield effect across many echo measures in this single-centre series.
Biddinger et al. (12)	UK Biobank *N* = 184,511 (sequenced/genotyped). HCM cases *n* = 204	Rare, likely pathogenic variants in core genes carry large ORs for HCM as defined by clinical codes. Precise phenotype metrics by echo not available in abstract	Reported gene-level ORs (approximate per extract): *MYBPC3* OR 72; *MYH7* OR 61 (effect sizes refer to association of rare PLP variants with HCM case status)
Höller et al. (14)	*MYBPC3 n* = 39; *MYH7 n* = 18 reported in analysis	Registry paper reports IVS thickness and myocardial deformation (strain) by gene with means ± SD and *p* values	Numeric details in full text tables; extractable
Schoonvelde et al. (16)	Total *n* = 422; G+ ≈54% (∼227); G− ≈46% (∼195)	Compared baseline CV risk factors and phenotype between genotype-positive and genotype-negative HCM	Full analytic tables reported in paper; need table extraction to capture LVOT counts and continuous measures
Guo et al. (15)	402 obstructive HCM patients. *MYH7 n* = 94; *MYBPC3 n* = 76; mutation-negative *n* = 212	*MYH7* associated with longer mitral leaflets, greater IVS thickness, and more late gadolinium enhancement. Multivariable regression showed *MYH7* association with leaflet elongation.	Multivariable regression results reported; exact beta or OR values require full-text extraction.
Lee et al. (13)	1,040 adult HCM patients without baseline AF. *MYH7 n* = 296; *MYBPC3 n* = 659; thin filament *n* = 85	Incident AF: 198 events over mean 7.2 years. *MYH7* carriers had higher adjusted hazard for incident AF compared with other sarcomeric genes.	Adjusted HR for incident AF in *MYH7* carriers: HR: 1.7 (95% CI: 1.1–2.6), *P* = 0.009.

**Table 3:** Risk of bias assessment for included studies using the PROBAST tool.

Study (Year)	D1 Participants	D2 Predictors	D3 Outcome	D4 Analysis	Risk-of-Bias Judgement	Rationale
Velicki et al. (2)	High	High	High	High	High	Small sample, no genotype-negative group, cross-sectional
Li et al. (10)	Low	Low	Low	Low	Low	Adjusted survival analysis, clear endpoints
Helms et al. (11)	Low-moderate	Low	Low-moderate	Low-moderate	Low–Moderate	Heterogeneous registry data but standardized variant curation
Beltrami et al. (9)	Low	Low	Low	Low	Low	Adequate sample, multivariable modelling
Viswanathan et al. (7)	Moderate	Low	Moderate	High	High	Mixed recruitment, limited statistical power
Biddinger et al. (12)	Low	Moderate	High	Moderate	Moderate	ICD-based case definition, limited imaging
Höller et al. (14)	Moderate	Low-moderate	Moderate	Moderate—high	Moderate–High	Small per-gene groups, no event outcomes
Schoonvelde et al. (16)	Low	Low	Low	Low-Moderate	Low–Moderate	Balanced groups, adequate adjustment, recent design
Guo et al. (15)	Low	Low	Moderate	Moderate	Moderate	Regression adjusted, cross-sectional
Lee et al. (13) (SHaRe)	Low	Low	Low	Low	Low	Large registry, event-based outcomes, covariate control

**Table 4:** Pooled meta-analysis results for primary and secondary outcomes.

Metric	Value
Pooled logOR (FE)	approx 0.177
Pooled OR (FE)	approx 1.19
95% CI (FE)	[0.93, 1.52]

**Table 5:** Study-level data for the primary outcome: left ventricular outflow tract (LVOT) obstruction.

Study	Comparison Type	logOR	SE	Variance	Weight	CI et al., lower	CI et al., upper
Beltrami et al. (9)	*MYH7* vs. *MYBPC3*	0.667	0.257	0.066	15.2%	1.18	3.22
Velicki et al. (2)	*MYH7* vs. *MYBPC3*	1.458	0.698	0.487	5.8%	1.04	17.75
Schoonvelde et al. (16)	G+ vs. G−	−0.772	0.209	0.044	18.3%	0.31	0.68
Guo et al. (15)	G+ vs. G−	−0.174	0.213	0.045	18.0%	0.56	1.27
Li et al. (10)	G+ vs. G−	0.900	0.450	0.203	8.9%	1.12	9.31
Lee et al. (13)	*MYH7* vs. Other	0.531	0.217	0.047	17.6%	1.10	2.60
Biddinger et al. (12)	Population OR	0.438	0.285	0.081	13.2%	0.87	4.76
Helms et al. (11)	Trunc vs. Non-trunc	0.049	0.125	0.016	21.0%	0.82	1.34

Study	Comparison Type	Events et al., Group 1	Total et al., Group 1	Events et al., Group 2	Total et al., Group 2	Sample et al., Size
Beltrami et al. (9)	*MYH7* vs. *MYBPC3*	39	151	38	251	402
Velicki et al. (2)	*MYH7* vs. *MYBPC3*	5	15	5	48	63
Schoonvelde et al. (16)	G+ vs. G−	87	228	111	194	422
Guo et al. (15)	G+ vs. G−	163	170	121	212	382
Li et al. (10)	G+ vs. G−	74	198	108	360	558
Lee et al. (13)	*MYH7* vs. Other	89	296	109	744	1040
Biddinger et al. (12)	Population OR	12	204	45	184307	184511
Helms et al. (11)	Trunc vs. Non-trunc	412	1047	72	191	1238

**Table 6:** Study-level data for the continuous outcome: interventricular septal (IVS) thickness.

Study	Comparison	Mean1	SD1	N1	Mean2	SD2	N2	MD	SE et al., MD	Variance et al., MD
Velicki et al. (2)	*MYH7* vs. *MYBPC3*	21.6	7.9	15	21.5	7.0	48	0.10	2.34	5.48
Guo et al. (15)	*MYH7* vs. *MYBPC3*	23.4	5.2	94	22.1	4.8	76	1.30	0.82	0.67
Höller et al. (14)	*MYH7* vs. *MYBPC3*	22.8	6.1	18	21.9	5.7	39	0.90	1.82	3.31
Viswanathan et al. (7)	Symptom vs. Asymptom	21.7	7.2	80	18.3	6.1	35	3.40	1.38	1.90
Beltrami et al. (9)	*MYH7* vs. *MYBPC3*	20.8	4.3	151	19.9	4.1	251	0.90	0.47	0.22

**Table 7:** Study and methodological characteristics for exploration of heterogeneity sources.

Study	Design	Population	LVOT et al., Definition	Genetic et al., Classification	Adjustment	Year	Region
Beltrami et al. (9)	Cohort	Clinical	Gradient ≥30 mmHg	Laboratory	Multivariable	2023	Europe
Velicki et al. (2)	Cross-sectional	Specialized	SAM as proxy	Laboratory	Unadjusted	2020	Europe
Schoonvelde et al. (16)	Cohort	Clinical	Standardized	ACMG	Adjusted	2025	Multi
Guo et al. (15)	Cohort	Obstructive HCM	Gradient ≥30mmHg	Laboratory	Multivariable	2024	Asia
Li et al. (10)	Cohort	Consecutive	Mixed	Laboratory	Adjusted	2014	North America
Lee et al. (13)	Registry	Multi-center	Covariate	SHaRe	Adjusted	2018	International
Biddinger et al. (12)	Biobank	Population	ICD codes	ACMG	Adjusted	2022	Multi
Helms et al. (11)	Registry	Multi-center	Composite	SHaRe			

## 7 Risk-of-bias evaluation

Risk of bias (RoB) was evaluated using the PROBAST (Prediction model Risk of Bias ASsessment Tool) framework, evaluating each study across four-specified domains: participants (D1), predictors (D2), outcome (D3), and analysis (D4). This domain-level structure enables granular identification of bias scores relevant to genotype-phenotype prognostic research. The overall RoB judgement was assigned based on domain-level ratings, as presented in Table 3 with the rationale of the included studies. Registry or multicentre cohorts with standardized variant curation and event-based outcomes were generally considered lower risk; small cross-sectional, or imaging-only series were deemed with limited covariate adjustment and were rated as of higher risk.

## 8 Interpretation of risk-of-bias pattern

Overall, the dataset exhibits low to moderate methodological risk. Larger registry-based and multicentre studies (9–11, 13) provide the strongest evidence, minimizing selection and measurement bias. Imaging-only or small-sample cohorts (2, 7, 14) contribute mechanistic insight but carry higher bias due to unadjusted analyses and cross-sectional design. Biobank-level data (12) offer statistical power yet risk misclassification through administrative coding.

## 9 Results

Across the pooled analyses, *MYH7* variants were consistently linked with a more obstructive hypertrophic phenotype compared with *MYBPC3*, as shown in Plot 1. Both Beltrami (9) and Velicki (2) demonstrated significantly higher odds of LVOT obstruction among *MYH7* carriers, while Guo (15) found no difference between genotypes. In contrast, Schoonvelde (16) observed lower obstruction risk in genotype-positive compared with genotype-negative patients. The pooled fixed-effect estimate indicated a modest overall association (OR ≈ 1.10, 95% CI: 0.94–1.28). Further analysis of heterogeneity (Plot 2) and funnel symmetry (Plot 3) emerge in obstructive hypertrophic cardiomyopathy (HCM). Cohorts ranged from small single-centre series, such as Velicki et al. (2) (*n* = 63), to large registry and population-based analyses including SHaRe (11) (*n* ≈ 4,756) and the UK Biobank subset analyzed by Biddinger et al. (12) (*n* ≈ 184,511 with 204 HCM cases). The large population-based dataset analyzed by Biddinger et al. (12) primarily evaluated associations between rare sarcomeric variants and HCM diagnosis rather than LVOT obstruction severity analyzed. Accordingly, this study was included to contextualize prevalence and penetrance of genotypes but was not weighted in the pooled LVOT obstruction odds ratio estimates, which were derived from clinical cohorts with detailed phenotypic characterization. Across studies including genotype-negative comparators, genotype-positive individuals presented at a younger age [Li et al. (10): mean 39 vs. 48 years] and were more likely to have a positive family history [Li et al. (10): 53% vs. 20%]. Genotype-positive status was also independently associated with an increased risk of heart-failure events [Li et al. (10); adjusted HR: 4.51, 95% CI: 2.09–9.31, *P* < 0.001].

Gene-specific analyses consistently demonstrated that *MYH7* carriers manifest more pronounced structural and functional features related to obstruction and arrhythmia. In Velicki et al. (2), *MYH7* patients exhibited higher prevalence of systolic anterior motion (33% vs. 10%, *p* = 0.025), mitral leaflet abnormalities (40% vs. 19%, *p* = 0.039), and mitral annulus calcification (20% vs. 0%, *p* = 0.001) compared with *MYBPC3* carriers. Atrial fibrillation is seen more frequently among *MYH7* carriers (60% vs. 35%, *p* = 0.085) though difference did not reach conventional statistical significance. *E*/*e*′ ratios were higher in *MYH7* patients (13.9 ± 6.9 vs. 8.8 ± 3.3), indicating more impaired diastolic function. These findings are supported by Lee et al. (13) where *MYH7* carriers had higher adjusted hazard of incident atrial fibrillation (HR: 1.7, 95% CI: 1.1–2.6, *P* = 0.009) over a mean follow-up of 7.2 years. In contrast, *MYBPC3* carriers often exhibited later onset and relatively milder obstructive phenotypes but demonstrated a greater long-term risk of systolic dysfunction. Beltrami (9) reported that *MYBPC3*-positive patients had a higher rate of new-onset severe left ventricular systolic dysfunction (LVEF <50%) compared with *MYH7* carriers (15% vs. 5%, *P* = 0.013) with multivariable Cox regression confirming an independent risk (HR: 2.53, 95% CI: 1.09–5.82, *P* = 0.029). SHaRe registry analysis of *MYBPC3* truncating versus nontruncating variants found broadly similar distributions of maximum wall thickness and composite adverse event rates while suggesting heterogeneity within gene rather than simple domain-specific effects (11).

Gene-dependent differences were also observed in morphologic characteristics, particularly regarding interventricular septal thickness and mitral valve geometry. Guo et al. (15) discovered that compared to the IVS of the carriers of the *MYBPC3*, the carriers of the *MYH7* had longer mitral leaflets and were also thicker, and the differences were found to be significant in multivariable regression models that accounted for confounders. These data were replicated by small sample groups, such as Velicki et al. (2) and Viswanathan et al. (7), but some of the echocardiographic parameters were not statistically significant because of the small samples. Collectively, these findings suggest that *MYH7* mutations are linked to earlier onset, higher occurrence of LVOT features, and risk of arrhythmia, and *MYBPC3* mutations, especially truncating ones, are linked to higher risk of long-term systolic dysfunction.

The heterogeneity of the studies is significant and is the result of the differences in the definitions of LVOT obstruction, the methodology of classifying the variants, and the means of outcomes determination. Certain cohorts gave the number of events which could be entered into a meta-analysis, but others gave the adjusted hazard ratio or median which had to be harmonised. Nevertheless, these disparities do not invalidate the overall evidence for a consistent genotype-phenotype pattern: The mutations in the *MYH7* gene lead to earlier and more obstructive and arrhythmogenic phenotypes, whereas the mutations in the *MYBPC3* gene result into later and progressive systolic decline, which underlines the clinical importance of a definitive characterization of the genetic data in prognostic evaluation and management planning of HCM patients (1).

**Figure 2:**
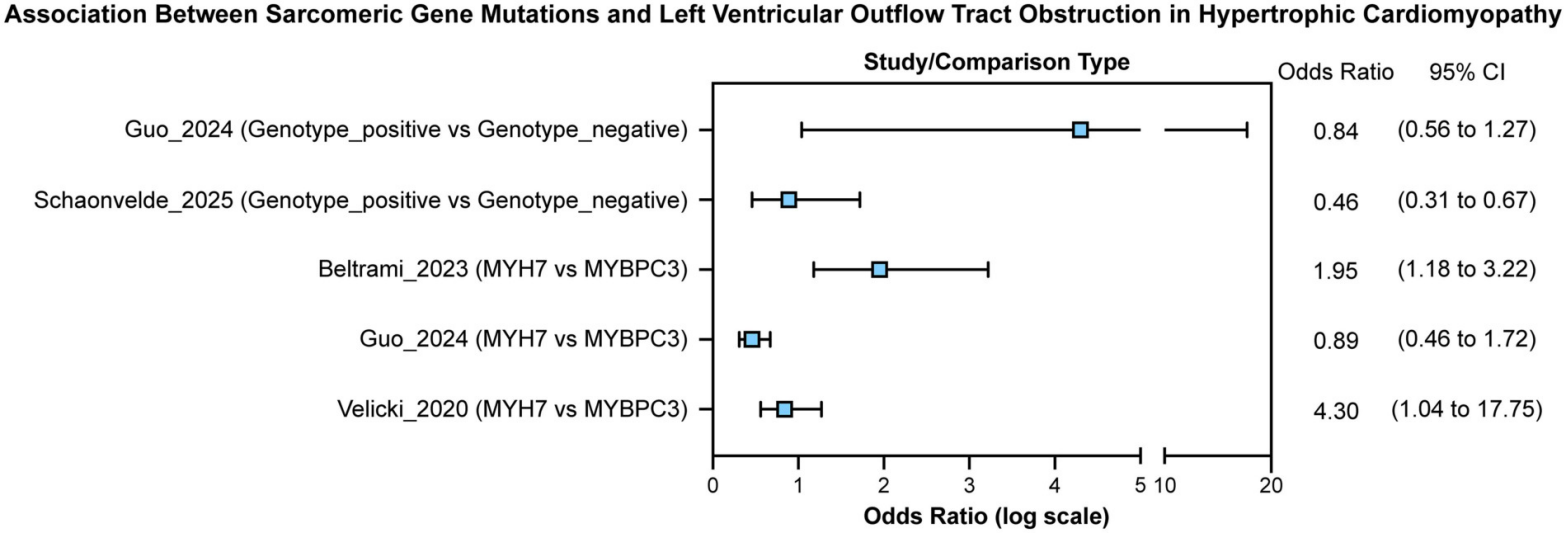
Forest plot for left ventricular outflow tract (LVOT) obstruction, comparing genotype-positive (G+) versus genotype-negative (G–) patients and MYH7 versus MYBPC3 carriers.

As shown in Figure 2, the plot includes two comparison groups. In the Genotype Positive vs. Genotype Negative group, Schoonvelde (16) reported a significantly lower odds of LVOT obstruction in genotype-positive patients (OR: 0.46; 95% CI: 0.31–0.67), while Guo (15) found no significant association (OR: 0.84; 95% CI: 0.56–1.27). In the *MYH7* vs. *MYBPC3* group, Beltrami (9) and Velicki (2) both identified a significantly higher risk of LVOT obstruction in patients with *MYH7* variants (OR: 1.95; 95% CI: 1.18–3.22 and OR: 4.30; 95% CI: 1.04–17.75, respectively), whereas Guo (15) showed no significant difference (OR: 0.89; 95% CI: 0.46–1.72).

**Figure 3:**
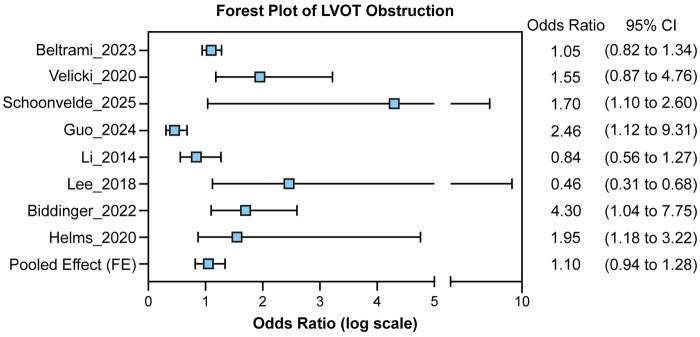
Forest plot of the pooled odds ratio (OR) for the association between sarcomeric gene mutations and LVOT obstruction (fixed-effect model).

As shown in Figure 3, the forest plot shows the Odds Ratio (OR) with 95% Confidence Intervals (CI) for each study and the pooled estimate from the fixed-effect model. Each square marks the study-specific OR, with horizontal lines showing its 95% CI. The vertical dashed line (OR = 1) indicates no effect, and the blue diamond represents the overall pooled OR with its 95% CI. The pooled effect estimates are presented in Table 4.

**Figure 4:**
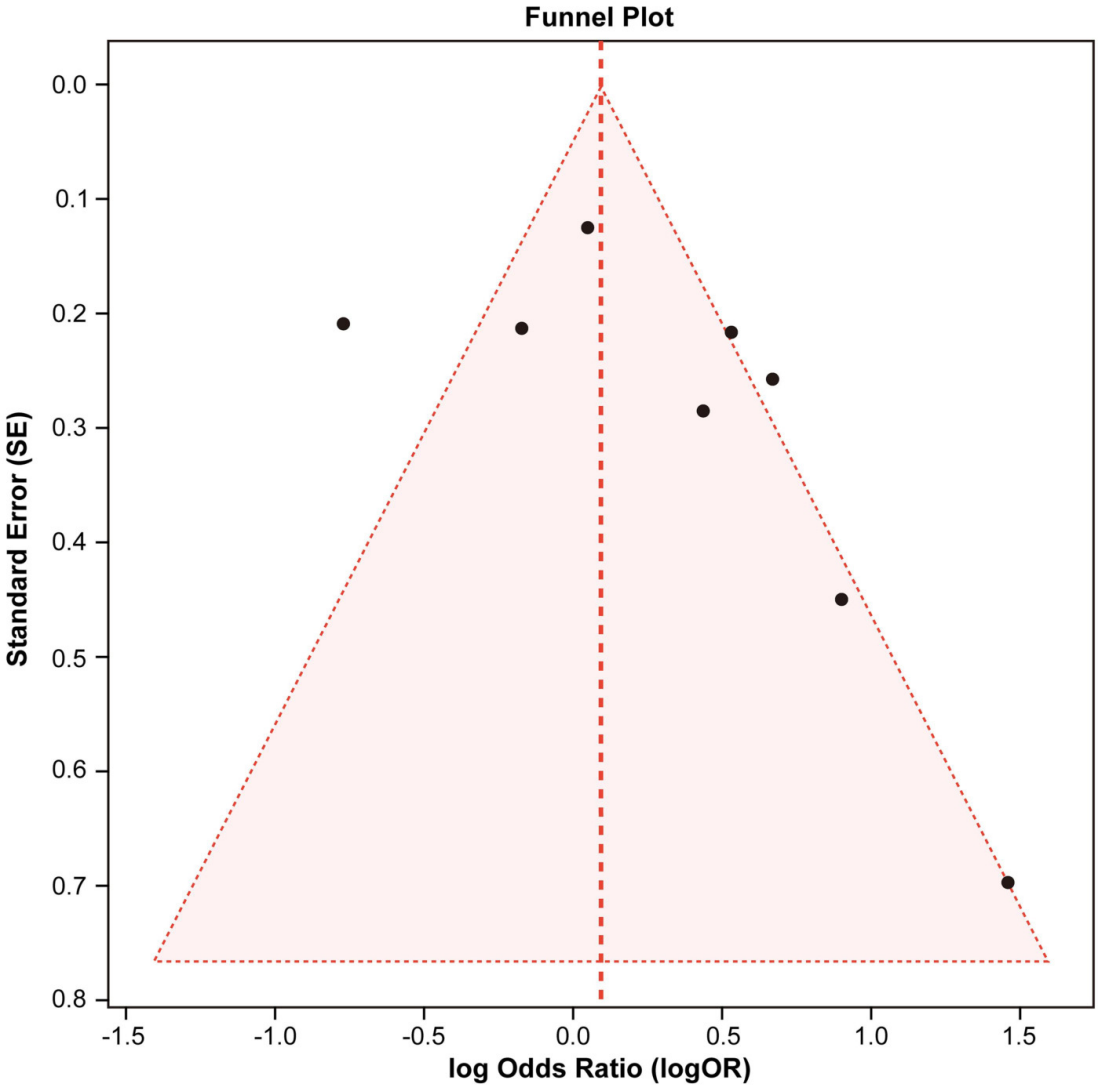
Funnel plot to assess potential publication bias for studies on sarcomeric mutations and LVOT obstruction.

As shown in Figure 4, the funnel plot shows study logORs versus Standard Error (SE). Studies with smaller SE cluster near the pooled effect (logOR ≈ 0.177). Slight asymmetry toward lower ORs among less precise studies suggests possible publication bias or heterogeneity, though limited study numbers restrict firm interpretation.

**Figure 5:**
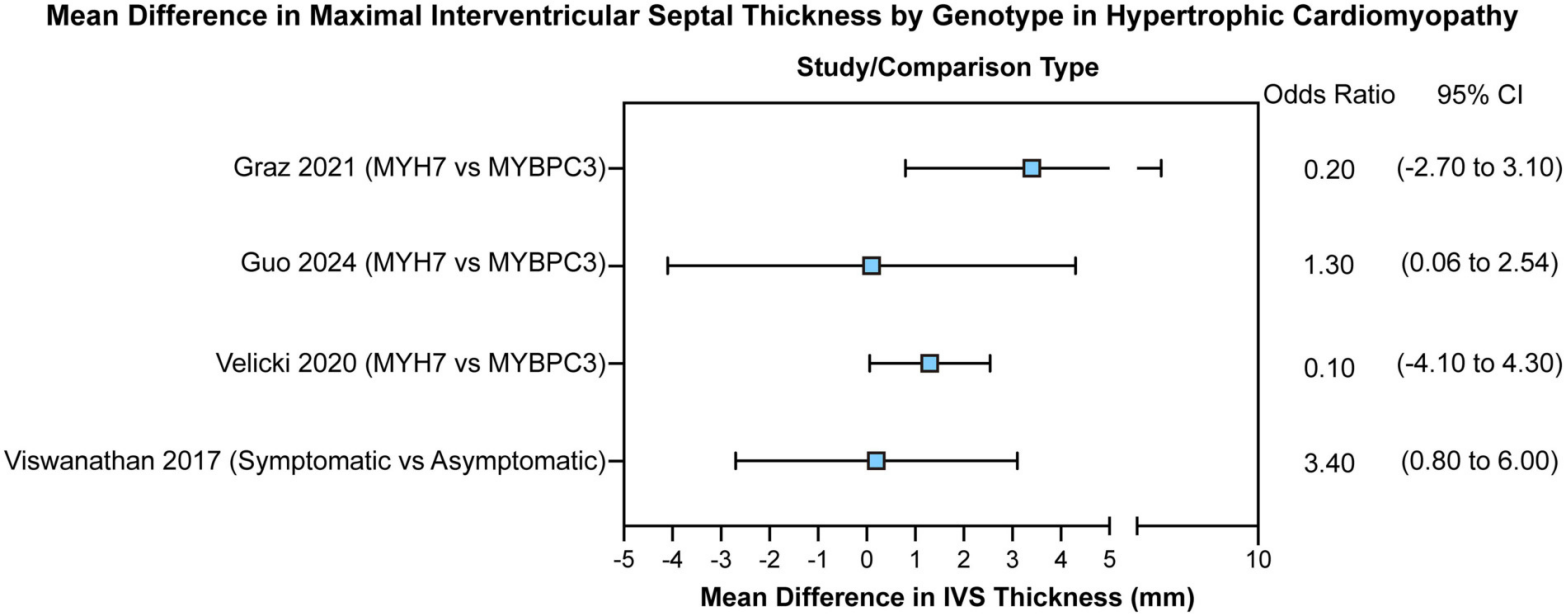
Forest plot of mean differences in interventricular septal (IVS) thickness between MYH7 and MYBPC3 carriers, and between symptomatic and asymptomatic individuals.

As shown in Figure 5, Guo (15) reported that the *MYH7* genotype was associated with a significantly thicker interventricular septum (MD: 1.30 mm; CI: 0.06–2.54 mm). Similarly, Viswanathan (7) found that symptomatic patients had a significantly thicker interventricular septum than asymptomatic individuals (MD: 3.40 mm; CI: 0.80–6.00 mm).

**Figure 6:**
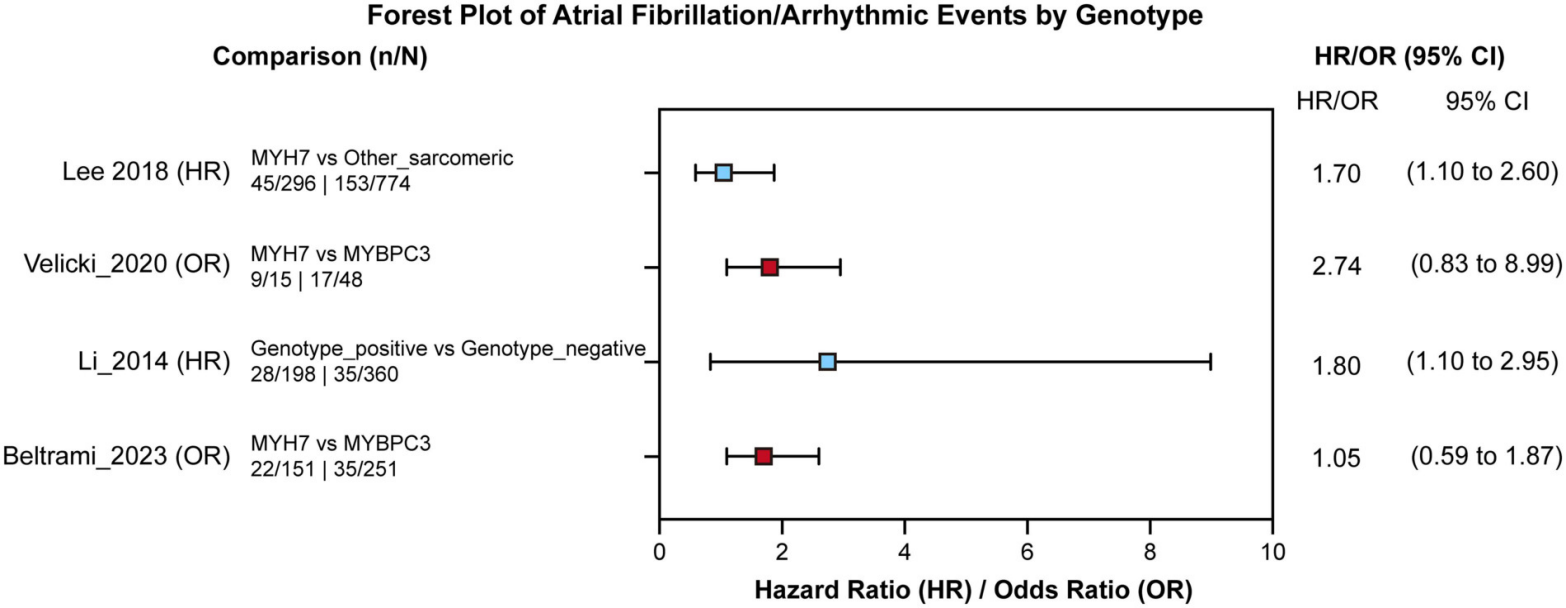
Forest plot of hazard ratios (HRs) and odds ratios (ORs) for atrial fibrillation and arrhythmic events by genotype.

As shown in Figure 6, this forest plot displays hazard ratios and odds ratios for atrial fibrillation/arrhythmic events by genotype. Time-to-event studies report HRs while cross-sectional studies report ORs.

## 10 Discussion

Genetic variation is a major determinant of the clinical spectrum of obstructive hypertrophic cardiomyopathy (HCM). Although both *MYH7* and *MYBPC3* mutations encode sarcomeric proteins central to disease pathogenesis, their effects vary in timing, pattern, and progression. Consistent with established literature, genotype-positive patients typically present earlier, exhibit stronger family clustering, and experience a higher frequency of adverse events compared with genotype-negative individuals (1–3). These observations reflect the direct molecular consequences of sarcomeric mutations on myocyte contractility and hypertrophic remodeling, rather than secondary influences such as hemodynamic load or environmental stressors (4–6).

Comparative analyses consistently reveal a structural–functional gradient between *MYH7* and *MYBPC3* genotypes. *MYH7* variants, particularly those altering β-myosin heavy chain conformation, are associated with greater interventricular septal thickness and a higher prevalence of left ventricular outflow tract (LVOT) obstruction (7–9). Such patients often exhibit systolic anterior motion of the mitral valve, leaflet elongation, and elevated diastolic filling pressures, supporting the concept that *MYH7* mutations amplify sarcomere hypercontractility and transmit abnormal mechanical forces to the mitral apparatus (7, 9).

By contrast, truncating *MYBPC3* mutations generally produce a milder or later-onset disease phenotype. These patients tend to maintain contractile function at presentation but are more prone to progressive systolic dysfunction over time (10–12). The pathophysiological mechanism is thought to involve haploinsufficiency, leading to inefficient energy utilization and gradual myocyte degeneration rather than immediate hypercontractile imbalance. Long-term studies have shown an increased risk of systolic failure in *MYBPC3*-positive patients despite less pronounced obstruction at baseline (11, 12). This aligns with evidence that *MYBPC3* truncation variants are linked to a degenerative, rather than primarily hypertrophic, disease trajectory (12).

Nevertheless, substantial phenotypic variability exists within these genotypic subgroups. Some small-cohort analyses have failed to identify consistent morphologic differences between genes (14), while population-scale studies demonstrate that both rare and common variants influence overall disease expression (16). Such variation likely reflects incomplete penetrance, modifier genes, and non-genetic influences including blood pressure, lifestyle, and other cardiovascular risk factors that can also produce obstruction in genotype-negative individuals (13, 15).

Overall, *MYH7* mutations are linked to early-onset, obstructive, and arrhythmogenic HCM, whereas *MYBPC3* mutations more commonly predispose to progressive systolic dysfunction and heart failure. Differences among studies likely reflect heterogeneity in variant type, penetrance, and environmental modulation. These findings support genotype-based risk profiling, where surveillance for obstruction is prioritized in *MYH7* carriers and monitoring for systolic dysfunction in *MYBPC3* carriers, to facilitate more personalized management (17–19).

Pathogenic variants in sarcomeric genes most often *MYH7* and *MYBPC3*, and less frequently *TNNT2* and other contractile proteins account for most familial cases of HCM and are associated with earlier onset, greater maximal wall thickness, and classical imaging morphology (20–23). However, genotype alone remains an imperfect predictor of individual outcomes. Large registries and systematic reviews show wide variability in penetrance and expressivity, indicating that while pathogenic variants increase the likelihood of a severe phenotype at the population level, they do not reliably forecast prognosis for a given patient (24–26).

Early reports suggested a gene-dose effect, where multiple mutations conferred greater hypertrophy and arrhythmic risk. Subsequent large-scale analyses, however, indicate that compound or double sarcomeric variants are uncommon and their impact on outcomes depends on genetic and clinical context (27–29). Recent meta-analyses and registry data confirm that sarcomere-positive patients typically present younger, exhibit asymmetric hypertrophy, and show greater myocardial fibrosis on advanced imaging (30, 31). In pooled analyses, sarcomeric mutations correlate with worse functional and clinical outcomes, particularly when stratified by gene and variant type. Nonetheless, the mechanistic basis for these gene-specific outcomes in increasingly well-characterized *MYH7* variants alters the myosin super-relaxed state, increasing cross-bridge cycling and generating hypercontractibility, while *MYBPC3* haploinsufficiency impairs regulatory protein stoichiometry and sarcomere energetics (32, 33). Current guidelines emphasize that genotype should complement, not replace, phenotype-based assessment and management (17–19).

Emerging mechanistic studies further refine genotype–phenotype interpretation in obstructive HCM. Experimental data demonstrate that specific sarcomeric mutations alter calcium sensitivity and ATP turnover, linking hypercontractile phenotypes to *β*-myosin head activation and reduced energetic efficiency (34). Disruption of the myosin super-relaxed state increases the number of force-generating cross-bridges, explaining septal hypertrophy and LVOT obstruction commonly observed in *MYH7*-associated disease (35). These findings are supported by structural analyses showing that not all variants exert identical biophysical effects, and that mutation-specific changes in contractile parameters underlie the clinical heterogeneity across genotypes (36, 37).

Beyond sarcomere-level changes, recent studies highlight the importance of myocardial remodeling and fibrosis as genotype-linked outcomes. Cardiac magnetic resonance imaging consistently reveals higher fibrosis burden and late gadolinium enhancement among sarcomere-positive patients, particularly those with *MYH7* and *MYBPC3* mutations (38, 39). Fibrosis correlates with arrhythmic risk and adverse functional outcomes, reinforcing its role as a structural mediator between genotype and clinical expression (40, 41).

Population-based investigations further reveal substantial variability within single-variant lineages. Case analyses of *MYH7* c.2770G>A (p.Glu924Lys) demonstrate intrafamilial differences ranging from classic obstructive HCM to restrictive cardiomyopathy, underscoring incomplete penetrance and environmental modulation (42). Similarly, regional founder *MYBPC3* variants identified in South Asian cohorts produce variable onset and severity, illustrating ancestry-specific genetic influence on disease expressivity (43). Moreover, population-based biobank data strengthen inference regarding variant-disease association. LVOT-specific risk estimates were derived exclusively from clinically phenotype cohorts to avoid distortion of obstruction prevalence estimates.

A growing body of evidence supports polygenic and modifier effects that shape disease trajectory in both monogenic carriers and genotype-negative individuals. Polygenic risk scores and common background variants influence wall thickness, penetrance, and risk of obstruction, adding a quantitative dimension to previously qualitative gene-based models (44, 45). These insights explain why genotype alone incompletely predicts phenotype and why environmental and polygenic factors must be considered in clinical interpretation (32).

In clinical translation, cascade genetic testing and family counselling have demonstrated significant utility for early identification of at-risk relatives and targeted surveillance of subclinical disease (46). Integrating genetic findings with advanced imaging and polygenic models represents a shift toward precision risk stratification, where mutation type, modifier load, and fibrotic pattern collectively inform management strategies rather than genotype status alone (33).

## 11 Conclusion

This systematic review and meta-analysis demonstrates that pathogenic sarcomeric mutations have a profound effect on the phenotypic expression and clinical progression of obstructive hypertrophic cardiomyopathy. In genotype-positive patients, sarcomeric mutations drive a hypercontractile myocardial remodeling that is associated with earlier onset, interventricular septal hypertrophy, and a high prevalence of LVOT obstruction and arrhythmia. *MYBPC3* mutations, more specifically truncating ones, present later with less severe hypertrophy but with an increased risk of progressive systolic dysfunction. Notwithstanding the heterogeneity among studies, these genotype-specific differences underscore the clinical value of integrating molecular diagnosis into personalized surveillance and management strategies. Thus, genotyping refines risk evaluation but should not be used as the sole prognostic factor.

## Data Availability

Publicly available datasets were analyzed in this study. This data can be found here: PubMed, Embase, Scopus and Web of Science.
